# Integrative Analysis for Elucidating Transcriptomics Landscapes of Glucocorticoid-Induced Osteoporosis

**DOI:** 10.3389/fcell.2020.00252

**Published:** 2020-04-16

**Authors:** Xiaoxia Ying, Xiyun Jin, Pingping Wang, Yuzhu He, Haomiao Zhang, Xiang Ren, Songling Chai, Wenqi Fu, Pengcheng Zhao, Chen Chen, Guowu Ma, Huiying Liu

**Affiliations:** ^1^School of Stomatology, Dalian Medical University, Dalian, China; ^2^School of Life Sciences and Technology, Harbin Institute of Technology, Harbin, China

**Keywords:** osteoporosis, glucocorticoid, microarray, protein-protein interaction, enrichment analysis

## Abstract

Osteoporosis is the most common bone metabolic disease, characterized by bone mass loss and bone microstructure changes due to unbalanced bone conversion, which increases bone fragility and fracture risk. Glucocorticoids are clinically used to treat a variety of diseases, including inflammation, cancer and autoimmune diseases. However, excess glucocorticoids can cause osteoporosis. Herein we performed an integrated analysis of two glucocorticoid-related microarray datasets. The WGCNA analysis identified 3 and 4 glucocorticoid-related gene modules, respectively. Differential expression analysis revealed 1047 and 844 differentially expressed genes in the two datasets. After integrating differentially expressed glucocorticoid-related genes, we found that most of the robust differentially expressed genes were up-regulated. Through protein-protein interaction analysis, we obtained 158 glucocorticoid-related candidate genes. Enrichment analysis showed that these genes are significantly enriched in the osteoporosis related pathways. Our results provided new insights into glucocorticoid-induced osteoporosis and potential candidate markers of osteoporosis.

## Introduction

Osteoporosis is the most common bone disease in the world, which is characterized by low bone mass, microstructure degeneration of bone tissue and decreased bone strength (Coughlan and Dockery, 2014). Osteoporosis is mainly divided into two categories, primary and secondary (Glaser and Kaplan, 1997). Primary osteoporosis further divided into three subtypes, postmenopausal osteoporosis, age-related osteoporosis and idiopathic osteoporosis. Secondary osteoporosis is a metabolic bone disease caused by disease or drugs, including glucocorticoid-induced osteoporosis. Excessive glucocorticoids can affect the differentiation and maturation of osteoblasts, leading to a decrease in their number and function, and can also promote the apoptosis of osteoblasts, thus further reduce bone formation (Whittier and Saag, 2016). Since the successful application of microarray technology, it has been widely used for expression profiling analysis in almost all fields of biological research (Sun et al., 2018). Due to its high-throughput characteristics, microarray technology has greatly advanced many areas of biological research by transforming the study of biology from a single gene level to the whole transcriptome-wide level (Canales et al., 2006; Cheng et al., 2019). Based on genome-wide microarray expression data, Xiao et al. (2008) studied osteoporosis-related B cells and emphasized the role of B cells in the pathogenesis of osteoporosis. Liu et al. (2005) compared gene expression in circulating monocytes from high and low bone mineral density samples based on the microarray data, revealing the role of monocytes in the pathophysiological mechanism of osteoporosis. In addition, some studies have focused on glucocorticoids and explored the mechanism by which they induce bone cell apoptosis (Lu et al., 2007; Jewell et al., 2012). However, these studies were only conducted in a limited number of single datasets one by one, and there was heterogeneity between different datasets. Therefore, more robust results will be obtained by integrative analysis of the multiple datasets.

In this study, we performed integrative analysis of two glucocorticoid-related osteosarcomas microarray datasets. Glucocorticoid-related gene modules were identified firstly. Then differential expression analysis was performed to obtain glucocorticoid-related differentially expressed genes in each dataset. In order to obtain robust results, the differentially expressed glucocorticoid-related genes of two datasets were intersected. Network analysis revealed that there were 158 robust glucocorticoid-related differentially expressed genes with interacting protein partners in protein-protein interaction network. Finally, gene function enrichment analysis showed that differentially expressed genes under glucocorticoid conditions were enriched in pathways associated with osteoporosis.

## Materials and Methods

### GEO Datasets

The glucocorticoid-related osteosarcoma U-2 OS bone cells microarray datasets of GSE6711 and GSE26857 were downloaded from Gene Expression Omnibus (GEO) database^1^ (Edgar et al., 2002), which included a total of 66 samples (48 glucocorticoid-treated and 18 untreated).

### Identification of Glucocorticoid-Related Gene Modules

Glucocorticoid-related gene modules of two datasets were identified by R package WGCNA, respectively (Langfelder and Horvath, 2008). The soft power applied for gene modules identification was 7 (GSE6711) and 24 (GSE26857). Correlation coefficients between the module Eigengenes and traits were calculated using Pearson’s method. Glucocorticoid-related gene modules were defined as those with correlation coefficients greater than 0.5 (Cheng et al., 2018).

### Identification of Differentially Expressed Genes (DEGs)

The DEGs of glucocorticoid-related gene modules were identified using the R package limma with a threshold of |log_2_FoldChange| >1 and *P* < 0.05. Then the genes up-regulated/down-regulated in both two datasets were considered as the robust up-regulated/down-regulated glucocorticoid-related DEGs.

### The Construction of Protein-Protein Interaction (PPI) Network

For the final up- or down-regulated glucocorticoid-related DEGs, we constructed PPI network by STRING database^2^, respectively (von Mering et al., 2003). Then Cytoscape (V.3.7.2) was used for network visualization (Shannon et al., 2003).

### Pathway Enrichment Analysis

We performed pathway enrichment analysis of DEGs included in PPI network using Enrichr^3^, an online enrichment analysis tool (Chen et al., 2013; Kuleshov et al., 2016). The significance threshold is *P* < 0.05.

## Results

### Identification of Glucocorticoid-Related Gene Modules

**FIGURE 1:**
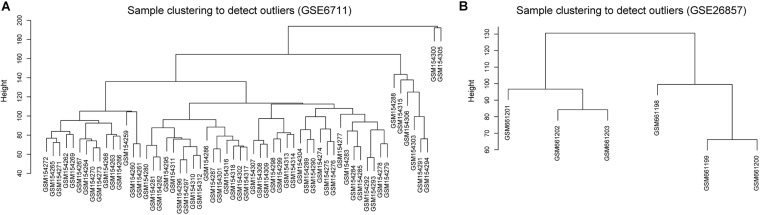
Hierarchical clustering of samples. **(A)** GSE6711 **(B)** GSE26857.

Glucocorticoid-induced osteoporosis is the most common form of secondary osteoporosis (Whittier and Saag, 2016; Coskun Benlidayi, 2018). To explore the correlation between glucocorticoid-induced gene expression disorder and osteoporosis, we collected two microarray datasets of glucocorticoid-related osteosarcoma cell lines from GEO database. The two datasets contained 66 samples, 48 of which were treated with glucocorticoids. According to the result of sample hierarchical clustering, we removed two outliers in GSE6711 (Figure 1).

**FIGURE 2:**
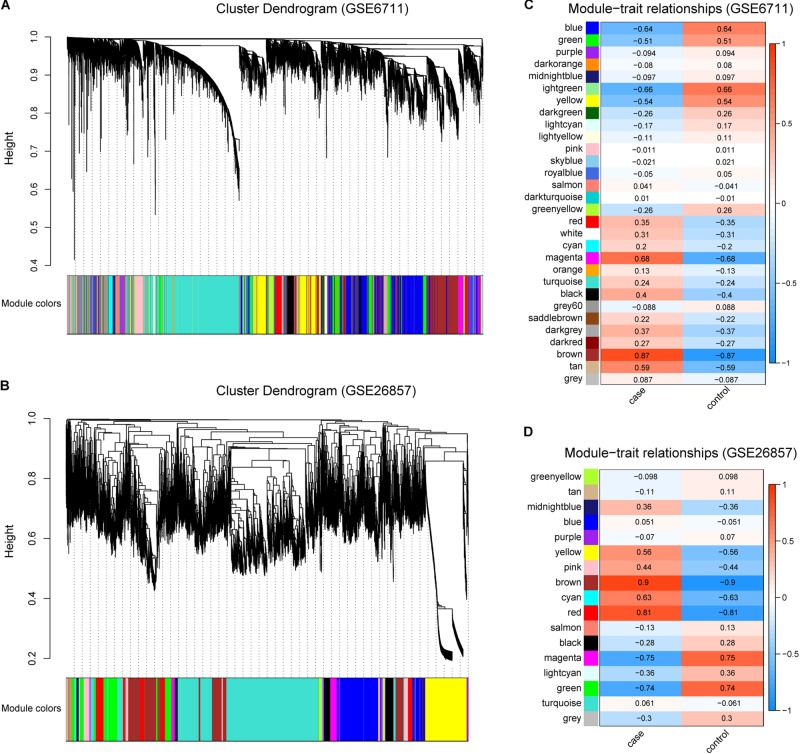
Identification of Glucocorticoid-related Gene Modules. **(A,B)** Gene modules identified by WGCNA in GSE6711 **(A)** and GSE26857 **(B)**. **(C,D)** The correlation between gene modules and trait in GSE6711 **(C)** and GSE26857 **(D)**, case represented glucocorticoid-treated and control represented untreated.

In order to obtain glucocorticoid-related genes, R package WGCNA was utilized. For all gene expression profiles, a total of 29 and 16 gene modules were identified in GSE6711 (Figure 2A) and GSE26857 (Figure 2B), respectively. The correlation between gene modules and traits suggested that three and four gene modules were correlated with glucocorticoids (*R* > 0.5, Figures 2C,D). Of these, brown was the most correlated module, followed by magenta module (GSE6711) and red module (GSE26857). In total, 3318 (GSE6711) and 5880 (GSE26857) genes were identified as glucocorticoid-related genes.

### Differential Expression Analysis of Glucocorticoid-Related Genes

**FIGURE 3:**
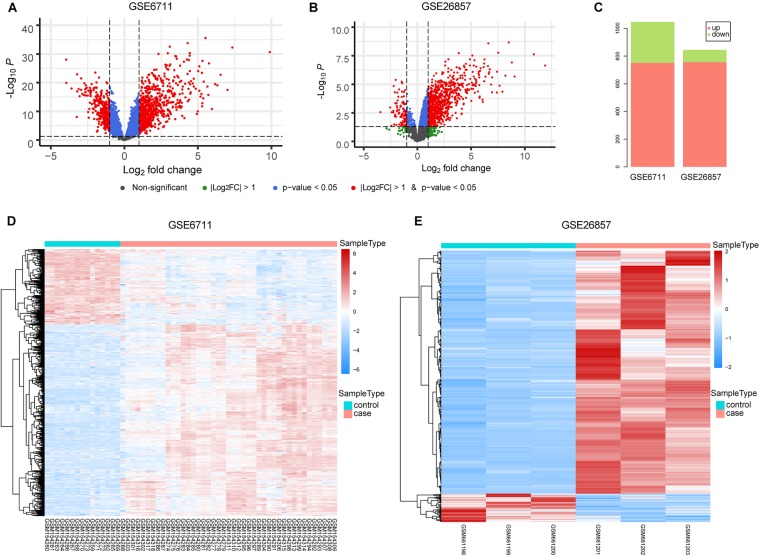
Differential Expression Analysis of Glucocorticoid-related Genes. **(A,B)** The volcano plot of glucocorticoid-related genes in GSE6711 **(A)** and GSE26857 **(B)**. **(C)** Barplot of differentially expressed glucocorticoid-related genes. **(D,E)** Heatmap of differentially expressed glucocorticoid-related gene expression profile.

The occurrence of diseases is often accompanied by gene expression disorders. We further analyzed the differential expression of glucocorticoid-related genes (Figures 3A,B). In GSE6711 dataset, 31.6% (1047/3318) genes were differentially expressed, including 751 up-regulated and 296 down-regulated (Figure 3C). In GSE26857 dataset, 14.4% (844/5880) genes were differentially expressed, including 756 up-regulated and 88 down-regulated (Figure 3C). It can be found that most of glucocorticoid-related DEGs were differentially up-regulated. Furthermore, the heatmap showed that DEGs were able to group the samples by sample types, which is glucocorticoid-treated (case) and untreated (control) (Figures 3D,E).

### Integration of Glucocorticoid-Related DEGs

**FIGURE 4:**
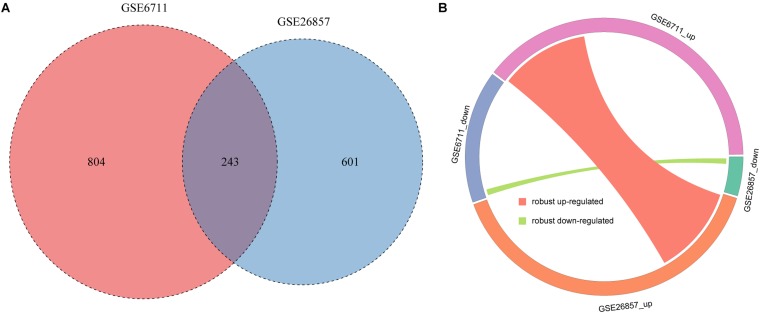
Integration of glucocorticoid-related DEGs. **(A)** The intersection of glucocorticoid-related DEGs in two datasets. **(B)** Circos plot of robust glucocorticoid-related DEGs.

Due to the data heterogeneity, there are some differences in the analysis results of different datasets. Therefore, integrative analysis of different datasets can get more robust results. We integrated the common glucocorticoid-related DEGs of two datasets. A total of 243 robust glucocorticoid-related DEGs were obtained, which accounted for 23.2 and 28.8% of glucocorticoid-related DEGs in two datasets (Figure 4A). Of the robust glucocorticoid-related DEGs, 242 genes were consistent in their deregulation directions between two datasets, including 227 up-regulated and 15 down-regulated genes (Figure 4B).

### Protein-Protein Interaction Network of Robust Glucocorticoid-Related DEGs

**FIGURE 5:**
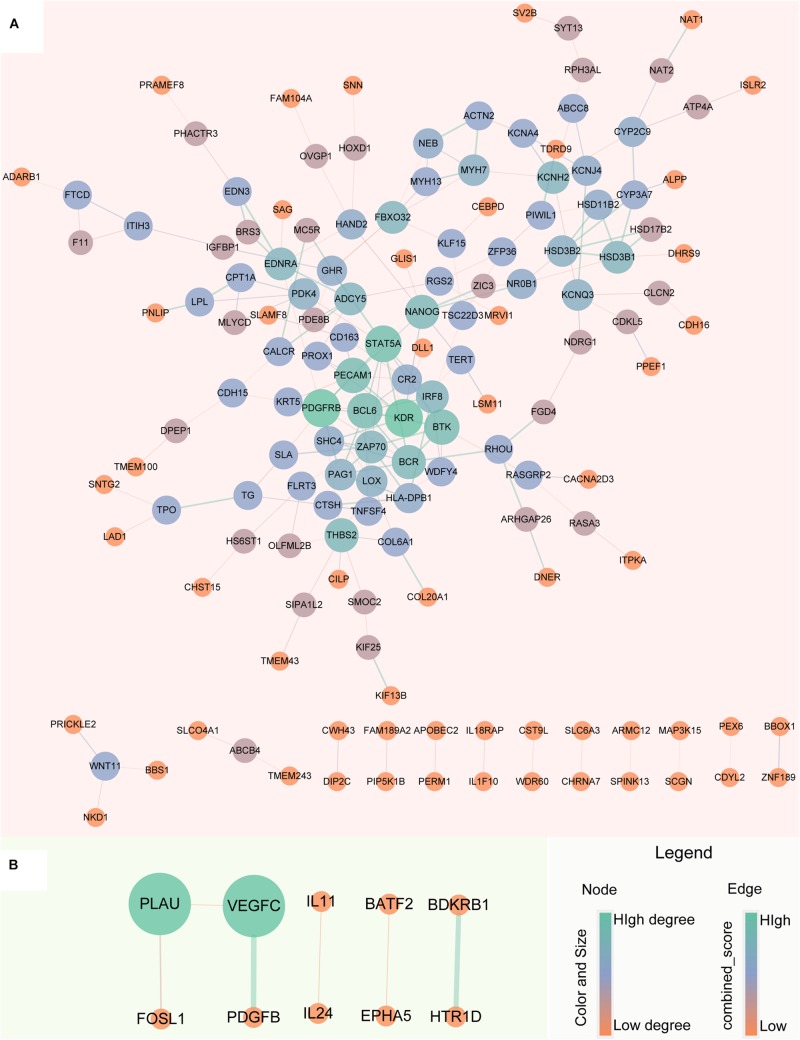
Protein-Protein Interaction network of robust glucocorticoid-related DEGs. **(A)** PPI network of robust up-regulated glucocorticoid-related DEGs. **(B)** PPI network of robust down-regulated glucocorticoid-related DEGs. The size and color of the node depends on the degree, the larger the degree, the larger the node. The width of the edge is determined by the combined score of STRING, the larger the score, the wider the edge.

The robust up-regulated and down-regulated DEGs were used to construct PPI networks, respectively. The results suggested that there are 148 up-regulated and 10 down-regulated DEGs have PPI relationship (Figure 5). The degree distribution of up-regulated DEGs PPI network ranges from 1 to 10 and the top 16 hub genes which degree greater than 5 can be found in Supplementary Figure S1.

**FIGURE 6:**
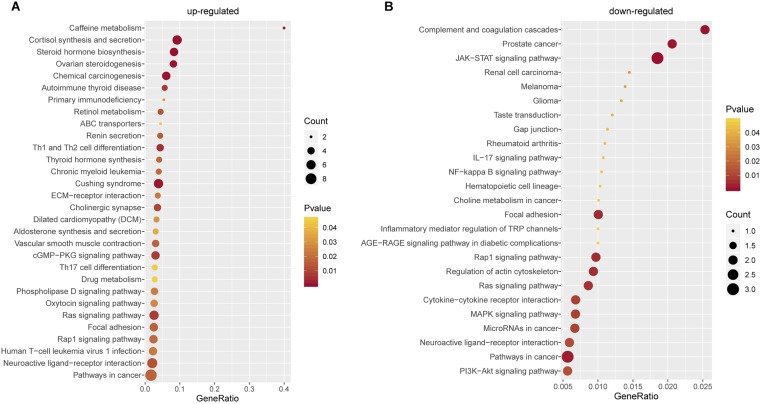
Pathway Enrichment Analysis. **(A)** Pathway enrichment analysis of up-regulated glucocorticoid-related DEGs in PPI network. **(B)** Pathway enrichment analysis of down-regulated glucocorticoid-related DEGs in PPI network.

To better understand the biological characteristics of robust glucocorticoid-related DEGs, we performed pathway enrichment analysis using up or down-regulated genes included in PPI networks by Enrichr, an online enrichment analysis tool. The up-regulated genes were significantly enriched in 30 KEGG pathways, including cushing syndrome, cortisol synthesis and secretion, Th1 and Th2 cell differentiation and Th17 cell differentiation (Figure 6A). Cushing’s syndrome is an endocrine disorder characterized by excessive cortisol secretion (Jiang et al., 2014; Pivonello et al., 2016). Many studies have shown that increased cortisol levels are associated with decreased bone mineral density (van Schoor et al., 2007; Osella et al., 2012; Toth and Grossman, 2013). The evidence for a relationship between the immune and the skeletal systems has long been recognized (Takayanagi, 2010; Yuan et al., 2010, 2011). Activated Th cells are important source of osteoclasts under inflammatory conditions (Rifas and Weitzmann, 2009). It is well known that osteoclasts are responsible for bone resorption and play an important role in osteoporosis (Detsch and Boccaccini, 2015; Kylmaoja et al., 2016; Zhang et al., 2020).

There were 25 pathways significantly enriched in down-regulated glucocorticoid-related DEGs (Figure 6B). Akt is the key element in osteoblast differentiation (Sugatani and Hruska, 2005). PI3K-Akt signaling pathway is one of the significant pathways, which can inhibit osteoporosis by promoting proliferation, differentiation and osteogenesis of osteoblasts (Xi et al., 2015). In addition, down-regulated genes were also enriched in multiple cancer-related pathways, it has been found that glucocorticoids play a role in the treatment of cancer (Pufall, 2015; Hu and Chen, 2017; McNamara et al., 2018).

## Discussion

In this study, we performed an integrative analysis of glucocorticoid-related microarray datasets. The WGCNA method enables the identification of glucocorticoid-related genes. Then differential expression analysis screened out the glucocorticoid-related dysregulated genes. By further integration, the robust dysregulated genes were obtained. Through protein-protein interaction network analysis of robust dysregulated genes, 158 candidate genes were obtained. And they significantly enriched the osteoporosis-related pathway.

In summary, we used microarray data to identify glucocorticoid-related dysregulated genes, which are associated with osteoporosis-related pathway. Our findings elucidate the expression mechanism of glucocorticoid-related genes and provide new guides for the diagnosis and treatment of glucocorticoid-induced osteoporosis.

## Data Availability Statement

Publicly available datasets were analyzed in this study. This data can be found here: The glucocorticoid-related osteosarcoma U-2 OS bone cells microarray datasets of GSE6711 and GSE26857 were downloaded from Gene Expression Omnibus (GEO) database (https://www.ncbi.nlm.nih.gov/geo/).

## Author Contributions

HL and GM designed the experiments. XJ obtained data from GEO. XY, XJ, PW, YH, HZ, XR, SC, WF, PZ, CC, and GM analyzed the data. XJ and HL wrote the manuscript. All authors read and approved the manuscript.

## Conflict of Interest

The authors declare that the research was conducted in the absence of any commercial or financial relationships that could be construed as a potential conflict of interest.

## Footnotes

1 https://www.ncbi.nlm.nih.gov/geo/

2 https://string-db.org/

3 http://amp.pharm.mssm.edu/Enrichr/

## Supplementary Material

FIGURE S1 The degree distribution of up-regulated DEGs PPI network.
