# Supplementary figures and images for: Integrative Analysis for Elucidating Transcriptomics Landscapes of Glucocorticoid-Induced Osteoporosis
Source: Front Cell Dev Biol. 2020 Apr 16;8:252. doi: 10.3389/fcell.2020.00252 (PMC7176994; doi:10.3389/fcell.2020.00252)

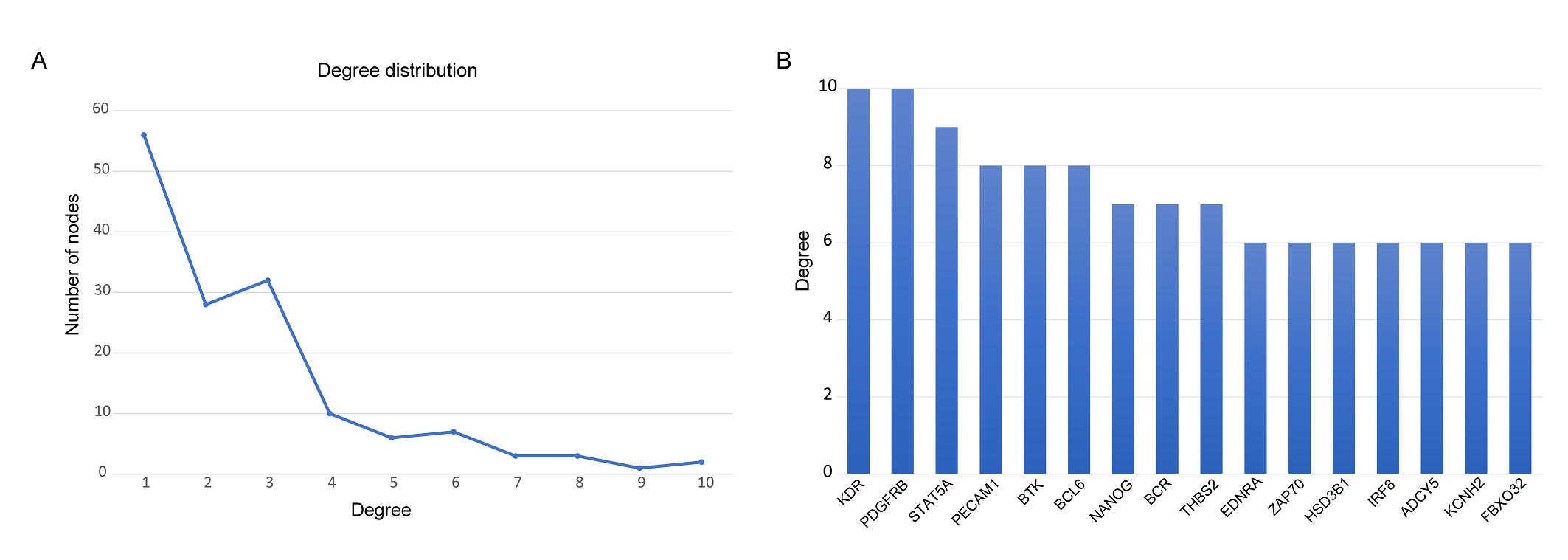

Supplement: FIGURE S1 — The degree distribution of up-regulated DEGs PPI network. [file Image_1.TIF]
